# Does yoga shape body, mind and spiritual health and happiness: Differences between yoga practitioners and college students

**DOI:** 10.4103/0973-6131.72630

**Published:** 2010

**Authors:** Elizabeth Monk-Turner, Charlie Turner

**Affiliations:** Department of Sociology, Old Dominion University, Norfolk, 23529 VA, USA; 1 Department of Economics, Old Dominion University, Norfolk, 23529 VA, USA

**Keywords:** College students, wellness, yoga students

## Abstract

**Background/Aims:**

To assess the body, mind and spirit differences between yoga students compared with college students.

**Materials and Methods:**

Mind, body and spirit survey instruments administered to the two groups.

**Results:**

Five indicators to measure mental wellness were significantly different between yoga practitioners and college students. On three of these five measures, college students reported more mental wellness than yoga practitioners – in other words, the relationship was the inverse of what was expected. College students reported maintaining stability in their life more often than yoga practitioners as well as more often experiencing satisfying interpersonal relationships. College students were also more likely than yoga practitioners to report being tolerant of others, whether or not they approved of their behavior or beliefs. Yoga practitioners were more likely than college students to report having strong morals and healthy values as well as the ability to express their feelings and consider the feelings of others. We found differences between yoga practitioners and college students on more than half of our spirit items (five of nine). Yoga practitioners were more likely than college students to report expressing their spirituality appropriately and in healthy ways, recognizing the positive contribution faith could make to the quality of life (significant at the 0.07 level), routinely undertaking new experiences to enhance spiritual health and having a positive outlook on life. Further, we found support for the proposition that yoga practitioners were more likely to report experiencing happiness within.

**Conclusions:**

Significant differences between yoga and college students were found on the body, mind and spirit measurement instrument. Further work needs to address the complexities of these relationships.

## INTRODUCTION

In the Yoga Sutras, yoga is defined to mean, “the yogic experience.” Yoga is often translated as “union” of mind, body and spirit. Classically, yoga is understood as the science of the mind. The yogic experience is that which is gained by controlling the modifications of the mind.[1] *Sri Patanjali*, considered the “father of yoga,” is credited with compiling the Yoga Sutras (the threads of yoga), which date anywhere from 5,000 B.C. to 300 A.D. In the West, yoga is primarily thought of as asanas (postures), breathing (*pranayama*) and meditation (*dhyana*).[2] It is estimated that 14.9 million Americans practice yoga and some suggest that yoga has become a transnational world practice.[3,4] Because many experience relaxation and ease with the practice of yoga, yoga is considered a mind-body exercise. The underlying premise of mind-body exercises is that the physiological state of the body may shape emotions, thoughts and attitudes.[5] In this work, we focus on differences in reported physical, mental, spiritual health and happiness and how this varies between yoga practitioners and a sample of college students.

### Defining and measuring wellness and happiness

We assess wellness by using a questionnaire based on the one developed by Hey, Calderon and Carroll.[6] This Body, Mind, Spirit Wellness and Characteristic Inventory (BMS) instrument is used to measure global health issues. How this questionnaire was constructed is detailed in our methodology.

Happiness is a difficult concept to define and measure. Most work carried out in this area allows individuals to self-define whether or not they are happy. One believes that they are happy or that they are not. A simple definition of happiness, from the Webster’s dictionary, is “good fortune” or “prosperity. A state of well-being and contentment: Joy.”[7] In the General Social Survey, like many other databases, when respondents are asked whether or not they are happy, happiness is self-defined. The respondent theoretically thinks “Am I happy”? and responds to the closed-ended option (yes/no) as appropriate. We too measure happiness in this way by asking respondents if they experience happiness within.

### Happiness research in psychology

Research on happiness has exploded in the past decade. Initially investigated by psychologists, who pioneered the field of positive psychology, and European economists, who reported that happiness is not critically linked to increased consumer consumption, today, it seems that many are interested in what factors shape happiness. In Authentic Happiness, Seligman outlines what he believes are the underlying conditions of happiness. Focusing on positive emotions (contentment, happiness, hope), character traits (love, courage, compassion, curiosity, integrity, moderation, to mention a few) and institutions (justice, responsibility, parenting), positive psychologists literally aim to make the world a happier place.[8] Seligman, who coined the term positive psychology, posits that individuals can change their mental state by altering how they perceive and react to events that occur in their lives. Seligman, past President of the American Psychological Association, has brought happiness research into the mainstream in the field of psychology. As the field matures, Seligman hopes to train clinicians to offer a nonpathological perspective in the field. Universities now offer classes in happiness, including Harvard University’s Ben-Shahar. Ben-Shahar has received widespread media attention for the popularity of his “Happiness 101” class. Prior to the advance of positive psychology, the discipline focused on pathology. Spurred by a growing body of research and general public interest, the Gallop Organization founded the Gallop Positive Psychology Institute (GPP). The GPP held its first meeting in 1999 and has witnessed significant interest and growth over time. In the US, Seligman is spearheading a drive to include a measure of well-being into national measures of the health of the US economy.

### Wellness and mindfulness research

Like psychology, the medical field as a whole is slowly evolving into a study of health as well as disease. More medical researchers are asking what makes for the health of the human organism rather than focusing solely on physical and mental problems. Many of these researchers challenge the traditional allopathic model of medicine – a paradigm that tends to posit a body–mind dichotomy and views bodily intelligence as resting in the mind. For example, Andrew Weil, a Harvard-trained physician, suggested in his 1983 book, Health and Healing, that the standard medical paradigm (viewing the human organism as a “complicated mechanism”; ignoring consciousness as a determinant of health; proclaiming itself scientific) fails to recognize that scientific reality has changed radically in the past century.[9] Weil recognizes the role of consciousness in the creation of health. Likewise, Christiane Northrup, past president of the American Holistic Medical Association, posits that the body is made of energy and is sustained by energy.[10] Thoughts are a part of this energy and, Northrup argues, have a well-documented effect on the body. Larry Dossey, whose work explores the power of prayer, maintains that the mind is “omnipresent, eternal and ultimately unitary or one.”[11] Deepak Chopra argues that there is no separation between body and mind. Rather, Chopra sees interdependence between one’s mental and physical health and the health of the society as a whole. One tool for creating health and wellness, Chopra believes, is meditation - the ultimate goal of many yoga practitioners.

Traditionally, in the West, a separation has been made between mind and body – a distinction attributed to the work of Descartes. Other medical traditions, like Traditional Chinese Medicine and Ayurveda, have long recognized the interconnectedness of body, mind and energy. Significant challenges to the biomedical model are emerging in medical science today. Health, wellness and happiness are perceived as legitimate subjects of medical and social investigation. In this work, we focus on differences in the reported physical, mental and spiritual health and happiness and how this varies between yoga practitioners and a sample of college students.

### Effects of yoga

A growing body of research explores yoga effects. This work typically focuses on the physical benefits of yoga. Still, yoga, as a practice, aims to integrate the body, mind and spirit. Thus, how the practice of yoga may shape measures of physical, mental, spiritual health and happiness is important to better understand, especially in light of the work reviewed above. Some work has reported that yoga asanas ease symptoms associated with osteoarthritis, carpal tunnel syndrome and low-back pain.[12–14] Low-back pain is one of the most likely reasons why people seek alternative health care.[15] Others have found that yoga improves cardiovascular health.[16,17] Reviewing the existing literature, Singh found that many scientific studies support the idea that yoga may be successfully used in treating essential hypertension, migraine, peptic ulcer, chronic sinusitis, intractable pain, anxiety, gastritis, bronchial asthma and headache, among others.[18,19] Others have argued that yoga is an effective system for weight loss and mild depression.[20,21]

Many researchers have found that yoga is effective for relieving stress and anxiety conditions that impact many physical and mental health conditions.[14] Especially, among those who reported mild to moderate levels of stress, researchers have found that practicing yoga significantly reduced anxiety.[22] Stress reduction programs have been conducted among healthy volunteers and among people with cancer, with data supporting the proposition that stress reduction decreases the cortisol levels.[23,24]

Yoga exercise that includes postures, breathing and meditation helps practitioners gain physical strength and flexibility as well as calm the mind.[25] In his work, Weil posits that correct breathing is critical to human health.[9] By adulthood, many have developed a pattern of restricted upper chest breathing. Hatha Yoga, the most widely practiced form of yoga in the West, encourages practitioners to learn various breathing exercises, including deep abdominal breathing, the three-part breath and lengthening the exhalation to mention a few.[26] Students of yoga frequently report a sense of deep relaxation, calm and happiness at the end of a yoga session. How well students of yoga may integrate this sense of relaxation and happiness into their lives is explored in this work.

Researchers have reviewed methodological issues related to yoga effects.[5,15] For example, some studies lack a control group. How yoga is defined frequently varies between studies. Some researchers look at the effects derived from the physical aspect of yoga while others rely mainly on the more meditative aspects of the practice. Still, we are persuaded that the evidence shows that yoga exercise is a practice that benefits overall health. Health is not a miracle or a stroke of luck.[25] While genetics shape health, our health is likewise conditioned by many factors that may be influenced, such as diet, exercise and stress-reduction techniques.

This work explores whether the practice of yoga, perhaps by reducing cortisol levels and stress thereby producing a feeling of well-being, is associated with increased levels of reported wellness and happiness. First, we examine whether there is a significant difference between yoga practitioners and college students along a number of items on the Body Mind Spirit Wellness Behavior Instrument developed by W.T. Hey and K.S. Calderon. We are especially interested in whether or not the reported levels of happiness differ between these two groups.

### Hypotheses

Because yoga is a body–mind exercise, we expect that yoga practitioners will be significantly different from others on variables related to the body, mind and spirit. Specifically, we expect yoga practitioners to report greater physical, mental and spiritual health, as measured by the BMS instrument, compared with a sample of college students. Further, we posit that yoga practitioners will be more likely to report that they experience happiness within as compared to others.

## MATERIALS AND METHODS

### Sample and data collection

Our sample comes from two sources. First, we compiled a sampling frame of all classes offered in two departments at a large urban university in the Spring of 2006. We selected Sociology and Criminal Justice and Exercise and Health Science classes. Exercise and Health Science classes were included because we wanted to highlight possible differences between yoga practitioners compared with college students, including college students who were studying on how to be healthy. Graduate, televised and off-campus classes were excluded from our sampling frame. Once the sampling frame was constructed, every fourth class was selected for the survey. Of course, before the project began, we obtained human subject approval from the College of Arts and Letters. In all, 135 students voluntarily completed the survey instrument. In addition, yoga practitioners who were taking hatha classes at a large yoga studio in the same urban area were surveyed. All classes offered at the studio were surveyed over a 2-week period. Given that this is a private studio, and that these classes were not introductory classes, we maintain that these students were serious yoga practitioners taking classes where teachers encourage a regular practice. Sixty-one yoga students completed the survey. Thus, our total sample consists of 196 respondents.

The BMS questionnaire aims to assess global health issues. Hey *et al*. maintain that this instrument is easy to administer and is capable of assessing wellness among college students.[6] They aim for others to use this instrument to assess BMS wellness in a variety of settings. Hey *et al*. reported that the reliability measures were fair to excellent for each dimension and ranged from 0.73 (Body) to 0.92 (Spirit) (between-scale correlations ranged from r=0.277 to r=0.526) (130). They maintain that this instrument is stable and that all subscales score in the same direction. Therefore, they argue that the instrument is a reliable and valid one.[6]

The BMS instrument includes 44 questions in three sections. There are nine questions relating to the body, 20 focused on the mind and another 15 questions about spirit. Our survey instrument excluded some questions from the BMS measure because we felt that some questions were repetitive and we aimed to shorten the survey instrument (see Table 1 for the BMS instrument that notes questions excluded). Thus, we have seven questions that focus on body, 14 that relate to the mind and nine that focus on spirit for a total of 30 questions. The two body questions we eliminated were: if the respondent used warm-up activities and if they ate a diet low in saturated fat. Warm-up activities were highly correlated with whether the respondent maintained their range of motion and if they had a reasonable amount of flexibility. Those who ate a variety of foods also ate a diet low in saturated fat. Again, the questions we excluded that focused on the mind were those that correlated highly with other questions. For example, we excluded the question “I learn from my mistakes,” but we did include the question “I learn from my past life experiences.” Finally, in the section on the spirit, we excluded questions on whether or not spirituality helped one remain calm and if one knew their purpose in life. However, we maintain questions that ask respondents if they experience harmony within or if they are content with who they are, and picked up information omitted from the excluded questions. Therefore, questions were eliminated if they were highly correlated with other questions as determined in a pretest among a small student sample. For each question, respondents were asked if they rarely/seldom (1), occasionally/sometimes (2) or often/always (3) engaged in this behavior (e.g., eating a balanced diet).

**Table 1:** Body mind spirit wellness behavior instrument developed and copyrighted by W.T. Hey and K.S. Calderon

1. Rarely/seldom 2. Occasionally/sometimes 3. Often/always
Body
I limit risky behaviors.
I maintain my fitness by exercising regularly and maintaining my weight.
I have a reasonable amount of flexibility and do exercises that help maintain my range of motion.
*I use warm-up activities before exercising to help prevent injuries.*
I eat a variety of foods and get the recommended number of servings from each food group.
*I eat a balanced diet low in saturated fat and cholesterol.*
I participate in recreational sports or activities that help maintain my fitness.
I drink at least eight glasses of water a day.
I surround myself with physically health people.
Mind
I learn from my past life experiences.
*I am open to new ideas.*
*I learn from my mistakes and try to behave differently the next time.*
*I talk with people rather than talk at people.*
I accept responsibility for my actions.
I understand and accept the existence of cultural diversity and its contribution to the quality of living.
I make good ethical decisions.
*I consider alternatives before making decisions.*
*I focus on reality.*
I am flexible to changes and can maintain stability in my life in healthy ways.
I have strong morals and healthy values.
I learn from the mistakes of others.
I have satisfying interpersonal relationships.
I feel loved and supported by family and friends.
I am tolerant of others whether or not I approve of their behavior or beliefs
I set achievable goals for myself.
*I handle various social settings well.*
I analyze my thoughts before I act.
I make the best of bad situations.
I express my feelings with others and consider their feelings.
Spirit
I experience harmony within.
*I experience peace of mind.*
I am in touch with the soul within.
I experience happiness within.
*I experience joy within.*
I experience self-satisfaction.
I express my spirituality appropriately and in healthy ways.
*My spirituality helps me remain calm and strong and helps me to better deal with difficult times*.
I recognize the positive contribution faith can make to the quality of my life.
I routinely undertake new experiences to enhance my spiritual health.
I have a positive outlook on life.
I am content with who I am.
*I know my purpose in life.*
*I read some form of spiritual literature on a regular basis.*
*I experience love of others and myself.*

(Items in italics used in our survey instrument)

### Analysis

All of our respondents were either college students or yoga practitioners. Thirty-one percent (61) of the sample consisted of yoga practitioners. Only nine students (out of 135) reported that they practiced yoga. These nine respondents were excluded from the analysis. More women (62%) were in our sample than men. The majority (65%) of the respondents were white (20% identified as African American/black). Among those practicing yoga, 77% were women (χ^2^=8.25; *P*=0.004). Significantly more women practiced yoga than men. Women were in a majority (56%) in our student sample as well. The vast majority (96%) of our students were between the ages of 18 and 34 years. Few (4%) were over 34 years old. Likewise, the majority (64%) of yoga practitioners were 18-34 years ols, while the remaining were over the age of 34 years. Finally, the vast majority (87%) of our yoga practitioners were identified as white, 3% as African American/black, 5% as Asian and 5% as of another race/background. Likewise, most (55%) of our student sample was white (more [28%] identified as African American/black). In regard to self-reported measures of physical, mental and spiritual health, our college sample has the advantage of age, of youth. Clearly, these samples are not matched in regard to gender, age or race. Given the very nature of these two groups, having two matched samples would be very difficult to identify.

### Physical wellness differences between yoga practitioners and college students

Of the seven questions asking respondents about their physical health, we found significant differences between yoga and college students in six [Table 2]. When asked if they maintained their fitness by exercising regularly and maintaining their weight, 71% of the yoga students reported that they often/always did so compared to only half of the college students (χ^2^=10.10; *P*=0.006). Yoga students were significantly more likely to report that they often/always did exercises to maintain their range of motion (77%) compared with only 39% of the college students (χ^2^=26.74; *P*=0.0001). Only 27% of the college students reported that they often/always got the recommended number of servings from each food group compared with 39% of the yoga students (χ^2^=6.12; *P*=0.04). Half (51%) of the yoga students said that they often/always participated in activities to help maintain fitness compared with 35% of the college students (χ^2^=9.77; *P*=0.007). Again, almost half (49%) of the yoga students said that they often/always drank at least eight glasses of water a day compared with only 24% of the college students (χ^2^=17.48; *P*=0.0002). Finally, yoga students were significantly more likely to report that they often/always surrounded themselves with physically healthy people (49% did so) compared with only 18% of the college students (χ^2^=22.87; *P*=0.0001). The only question that yoga and college students did not significantly differ on with regard to body measures was how often they limited risky behaviors. Data support our hypothesis that yoga practitioners would report healthier physical choices compared with a sample of college students.

**Table 2:** Body mind spirit wellness behavior instrument (restricted questions). Chi-square values between a sample of yoga practitioners and college students

Chi-square value *P*-value Body		
I limit risky behaviors	NS	
I maintain my fitness by exercising regularly and maintaining my weight	10.10	0.006
I have a reasonable amount of flexibility and do exercises that help maintain my range of motion	26.74	0.0001
I eat a variety of foods and get the recommended number of servings from each food group	6.12	0.04
I participate in recreational sports or activities that help maintain my fitness	9.77	0.007
I drink at least eight glasses of water a day	17.48	0.0002
I surround myself with physically health people	22.87	0.0001
Mind		
I learn from my past life experiences	NS	
I accept responsibility for my actions	NS	
I understand and accept the existence of cultural diversity and its contribution to the quality of living	NS	
I make good ethical decisions	NS	
I am flexible to changes and can maintain stability in my life in healthy ways*	5.86	0.05
I have strong morals and healthy values	10.83	0.004
I learn from the mistakes of others	NS	
I have satisfying interpersonal relationships*	5.40	0.06
I feel loved and supported by family and friends	NS	
I am tolerant of others whether or not I approve of their behavior or beliefs*	7.16	0.03
I set achievable goals for myself	NS	
I analyze my thoughts before I act	NS	
I make the best of bad situations	NS	
I express my feelings with others and consider their feelings	7.14	0.03
Spirit		
I experience harmony within	NS	
I am in touch with the soul within	NS	
I experience happiness within	5.25	0.07
I experience self-satisfaction	NS	
I express my spirituality appropriately and in healthy ways	7.04	0.03
I recognize the positive contribution faith can make to the quality of my life	5.27	0.07
I routinely undertake new experiences to enhance my spiritual health	7.96	0.02
I have a positive outlook on life	12.19	0.002
I am content with who I am		NS

* Significant, but the relationship is opposite of that hypothesized, NS=Not significant

### Mental wellness differences between yoga practitioners and college students

We asked 14 of the original 20 BMS questions that centered on the mind. Of these 14 questions, we found significant differences between yoga practitioners and college students on only four measures. Further, for two of these four items, college students reported more mental wellness than yoga practitioners (contrary to the hypothesized direction) (Table 2 summarizes these results).

The vast majority (70%) of college students reported that they were often/always flexible to changes and maintained stability in their life in healthy ways compared with 54% of the yoga practitioners (χ^2^=5.86; *P*=0.05). When asked if they were tolerant of others regardless of differences in behaviors or beliefs, again significantly more (74%) college students than yoga practitioners (57%) reported that they often/always felt this way (χ^2^=7.16; *P*=0.03).

The vast majority (77%) of yoga practitioners felt that they often/always had strong morals and healthy values compared with 56% of college students (χ^2^=10.83; *P*=0.004). Further, more yoga practitioners (66%) reported feeling that they often/always expressed their feelings with others and considered their feelings compared with 45% of college students (χ^2^=7.14; *P*=0.03).

In summary, more yoga practitioners than college students reported that they had a strong sense of morals/values and that they more often expressed their feelings with others. However, college students were more likely than yoga practitioners to report that they often/always maintained stability in their life in a healthy way, or that they were tolerant of others. Yoga practitioners tended to be split more equally on these measures between those who reported occasionally/sometimes feeling this way or often/always feeling this way. We found no significant differences between yoga practitioners and college students on most mind measures, including learning from past life experiences, accepting responsibility for one’s actions, having satisfying interpersonal relationships, valuing cultural diversity, making good ethical decisions, learning from the mistakes of others, feeling loved, setting achievable goals, analyzing thoughts before acting or making the best of bad situations.

### Spiritual wellness and happiness differences between yoga practitioners and college students

Of our nine measures of spiritual health, we found significant differences between yoga practitioners and college students on four items. Significantly more yoga practitioners (57%) felt that they expressed their spirituality appropriately and in a healthy way compared with only 38% of college students (χ^2^=7.04; *P*=0.03). Likewise, yoga practitioners (61%) were more likely than college students (43%) to feel that they often times recognized the positive contribution faith could make to the quality of life (χ^2^=5.27; *P*=0.07). More college students (19%) than yoga practitioners (8%) reported that they rarely undertook new experiences to enhance their spiritual health (χ^2^=7.96; *P*=0.02). Finally, more yoga practitioners (67%) than college students (42%) reported that then often times had a positive outlook on life (χ^2^=12.19; *P*=0.002).

We are especially interested in whether or not reported happiness differed between yoga practitioners and college students. Only 3% of the yoga practitioners said that they rarely felt happiness within compared to 12% of the college students. More yoga practitioners (97%) reported that they at least occasionally felt happiness within compared to 88% of college students (χ^2^=5.25; *P*=0.07). This difference nears statistical significance at the 0.05 level; therefore, we suggest that yoga may shape the feeling of happiness within compared to those who do not practice yoga.

Yoga practitioners were more likely than college students to report that they often times experienced happiness within, expressed their spirituality appropriately and in healthy ways, recognized the positive contribution faith could make in ones life, undertook new experiences to enhance spiritual health or had a positive outlook on life. Again, we did not find significant differences between yoga practitioners and others on measures of experiencing harmony within, being in touch with the soul within, experiencing self-satisfaction or being content with who they were.

## DISCUSSION

As expected, yoga practitioners were significantly more likely than college students to report at least occasionally making healthy choices for the physical body than rarely or seldom doing so. Yoga practitioners reported engaging in such behaviors significantly more often than college students on all but one measure that asked about limiting risky behaviors.

Only five indicators to measure mental wellness were significantly different between yoga practitioners and college students. On three of these five measures, college students reported more mental wellness than yoga practitioners–in other words, the relationship was the inverse of what was expected. College students reported maintaining stability in their life more often than yoga practitioners as well as more often experiencing satisfying interpersonal relationships. One might argue that yoga practitioners were more sensitive to the quality of stability or relationships in their lives, leading to being less satisfied along these measures than college students. Alternatively, one could maintain that a search for more stability, and finding satisfying interpersonal relationships, might lead one into the practice of yoga. Clearly, we do not have the data here to sort out these issues. College students were also more likely than yoga practitioners to report being tolerant of others whether or not they approved of their behavior or beliefs. Given that this item included reported tolerance for either behavior or beliefs, it is impossible to sort out whether this difference would hold if these two concepts were separated. Nevertheless, the goal of being tolerant of others, accepting differences, is lacking among yoga practitioners in our sample. Are yoga practitioners less tolerant because they believe they are on a superior path or might they just be less tolerant and forgiving of themselves? Alternatively, the college environment encourages students to respect others despite differences. Again, given our data, we cannot address what factors shape this relationship between tolerance differences between yoga practitioners and college students.

Yoga practitioners were more likely than college students to report having strong morals and healthy values as well as the ability to express their feelings and consider the feelings of others. Yoga teachers are typically trained about yamas (abstinence) and niyamas (observances) – two of the eight limbs of ashtanga yoga. Yamas include the goal of nonviolence, truthfulness, nonstealing, nonsensuality and nongreed. Niyamas (ni means without) refer to behaviors that need no restraints and aim for purity, contentment, austerity, self-study and attunement to life. Many yoga teachers include these ideas as part of their instruction. Perhaps it is not surprising that yoga practitioners are more likely than college students to report having strong morals, healthy values and the ability to more often express their feelings.

We found differences between yoga practitioners and college students on more than half of our spirit items (five of nine). Yoga practitioners were more likely than college students to report expressing their spirituality appropriately and in healthy ways, recognizing the positive contribution faith could make to the quality of life (significant at the 0.07 level), routinely undertaking new experiences to enhance spiritual health and having a positive outlook on life. We were especially interested in whether or not yoga practitioners would report experiencing happiness within significantly more often than college students. We found support for the proposition that yoga practitioners were more likely to report experiencing happiness within (at the 0.07 level) compared to college students.
